# Donor NKG2A/NKG2C Immunophenotype Influences the GMP-Compliant Manufacturing Potential of NK Cells for Adoptive Immunotherapy

**DOI:** 10.3390/cancers18172732

**Published:** 2026-08-23

**Authors:** Rut Meseguer, Cristobal Aguilar, Paula Amat, Luis Larrea, Ana Bonora, Pedro Chorão, Francisco Boix, Jose Luis Piñana, Rosa Guerrero, Pau Montesinos, Belén Vera, Dolores Planelles, Mar Luis, Jose Luis Poveda, Javier De la Rubia, Sergi Querol, Cristina Arbona, Manuel Guerreiro, Carlos Solano

**Affiliations:** 1 Hematology Service, Hospital Clínico Universitario, Health Research Institute INCLIVA, 46010 Valencia, Spain; rmeseguer@incliva.es (R.M.); amat_pau@gva.es (P.A.); pinana_jos@gva.es (J.L.P.); 2 Advanced Therapies Unit, Health Research Institute La Fe, 46026 Valencia, Spain; cristobal_aguilar@iislafe.es (C.A.); ana_bonora@iislafe.es (A.B.); 3 Medicine Department, University of Valencia, 46010 Valencia, Spain; 4 Transfusion Centre of the Valencian Community, 46014 Valencia, Spain; larrea_lui@gva.es (L.L.); boix_fragin@gva.es (F.B.); guerrero_ros@gva.es (R.G.); vera_bel@gva.es (B.V.); planelles_dol@gva.es (D.P.); luis_marhid@gva.es (M.L.); arbona_cri@gva.es (C.A.); 5 Hematology Service, Hospital Universitario y Politécnico La Fe, 46026 Valencia, Spain; chorao_ped@gva.es (P.C.); montesinos_pau@gva.es (P.M.); delarubia_jav@gva.es (J.D.l.R.); 6 Centro de Investigación Biomédica en Red de Cáncer (CIBERONC), 28029 Madrid, Spain; 7 Hematology Research Group, Health Research Institute La Fe, 46026 Valencia, Spain; manuel_direito@iislafe.es; 8 Pharmacy Department, Hospital Universitario y Politécnico La Fe, 46026 Valencia, Spain; poveda_josand@gva.es; 9 Pharmacy Research Group, Health Research Institute La Fe, 46026 Valencia, Spain; 10 Fundación Internacional Josep Carreras, 08021 Barcelona, Spain; sergi.querol@fcarreras.es

**Keywords:** adaptive NK cells, natural killer cells, NKG2A, NKG2C, allogeneic hematopoietic stem cell transplantation, GMP manufacturing, adoptive immunotherapy, cytomegalovirus

## Abstract

**Background:** Relapse and opportunistic viral infections remain major causes of treatment failure after allogeneic hematopoietic stem cell transplantation (allo-HSCT). Adoptive transfer of natural killer (NK) cells is a promising strategy to enhance post-transplant immune reconstitution. Adaptive NKG2C+ NK cells exhibit enhanced cytotoxicity and persistence, but the influence of baseline donor immunophenotype on GMP manufacturing has not been systematically investigated. We evaluated whether donor NKG2A/NKG2C immunophenotypes are associated with successful manufacture of adaptive NK-cell products. **Methods:** Eighty-three healthy donors from the ReDoCel registry underwent immunophenotypic characterization of circulating NK-cell subsets by multiparametric flow cytometry. Donors were stratified by unsupervised hierarchical clustering according to NKG2A/NKG2C expression. Representative donors from NKG2C- and NKG2A-dominant clusters underwent feeder-free GMP-compliant expansion using the automated CliniMACS Prodigy® platform (Miltenyi Biotec, Bergisch Gladbach, Germany). Expanded products were evaluated for manufacturing efficiency, immunophenotype, cytotoxic function, and post-thaw stability. **Results:** Baseline NKG2C frequencies showed marked inter-donor variability, allowing identification of four immunophenotypic clusters. Only the NKG2C-dominant donor achieved successful GMP manufacturing, exceeding the predefined expansion threshold while maintaining high viability and purity. Both NKG2A-dominant donors showed limited proliferative capacity under identical manufacturing conditions. Expanded NK cells acquired an activated phenotype characterized by increased expression of DNAM-1, NKG2D, NKp30, NKp46, and TIM-3 while preserving mature differentiation and KIR expression. Functional analyses demonstrated potent degranulation against leukemia targets with minimal autoreactivity, resulting in a predominantly cytotoxic effector profile. The successfully expanded product maintained viability, phenotype, and function after long-term cryopreservation. **Conclusions:** This proof-of-concept study suggests that baseline donor NKG2A/NKG2C immunophenotype may influence GMP manufacturing of adaptive NK-cell products. These findings support prospective evaluation of donor immunophenotyping as a biomarker for donor qualification and manufacturing optimization to facilitate standardized off-the-shelf adaptive NK-cell therapies after allo-HSCT.

## Simple Summary

Natural killer cells are promising candidates for cellular therapy after allogeneic hematopoietic stem cell transplantation because they can help control malignant cells and viral infections with a low risk of graft-versus-host disease. However, the ability to manufacture these cells consistently under good manufacturing practice conditions may vary between donors, and donor-related factors are not well understood. In this proof-of-concept study, we investigated whether the baseline distribution of two natural killer cell receptors, NKG2A and NKG2C, was associated with successful cell manufacturing. We characterized 83 healthy donors and selected three donors with different receptor profiles for manufacturing using an automated closed system. Only the donor enriched in NKG2C-positive cells achieved the predefined manufacturing criteria. These findings suggest that donor immunophenotyping may be a promising factor to consider when evaluating donor suitability for natural killer cell manufacturing and warrants validation in larger studies.

## 1. Introduction

Allogeneic hematopoietic stem cell transplantation (allo-HSCT) is the standard curative treatment for a broad range of high-risk hematological malignancies and selected non-malignant disorders. It is most commonly performed for patients with acute myeloid leukemia (AML) and myelodysplastic syndromes (MDSs) but is also indicated for a variety of other hematological disorders [1,2]. The therapeutic efficacy of allo-HSCT relies not only on the cytoreductive effects of the conditioning regimen but also on the graft-versus-leukemia (GVL) effect mediated by donor-derived immune cells, which is essential for durable disease control. Despite major advances in transplantation procedures and supportive care, disease relapse remains the leading cause of treatment failure, while graft-versus-host disease (GVHD) and opportunistic infections, including viral reactivations, continue to contribute substantially to post-transplant morbidity and mortality [2].

Following allo-HSCT, natural killer (NK) cells are among the first lymphocyte populations to reconstitute, typically within the first weeks after transplantation, preceding T-cell recovery [3]. This rapid immune reconstitution provides a unique opportunity to exploit NK cells for early post-transplant adoptive immunotherapy.

NK cells are innate lymphocytes that play a central role in immune surveillance against malignant transformation and viral infections. Unlike T lymphocytes, they recognize and eliminate target cells independently of major histocompatibility complex class I (MHC-I), enabling rapid cytotoxic responses with minimal risk of GVHD [4,5]. Their activity is regulated by the balance between activating and inhibitory germline-encoded receptors, including killer-cell immunoglobulin-like receptors (KIRs), NKG2A, and NKG2C [6]. Upon activation, NK cells mediate cytotoxicity through perforin- and granzyme-dependent mechanisms, antibody-dependent cellular cytotoxicity (ADCC), and cytokine secretion, thereby contributing to both antitumor and antiviral immunity [7,8,9,10].

The favorable safety profile of NK cells, together with their potent antitumor and antiviral activity, has positioned NK-cell adoptive immunotherapy as a promising strategy to improve immune reconstitution after allo-HSCT. Recent studies have highlighted that cellular immunotherapies administered during the early post-transplant period may exploit the low tumor burden and the permissive immune environment to enhance immune reconstitution while reducing the risk of relapse and opportunistic infections [11]. In this setting, the development of readily available, GMP-compliant off-the-shelf immune-cell products has become a key translational goal, driving efforts to optimize both manufacturing platforms and donor selection strategies [11,12]. Clinical studies have demonstrated that donor-derived and third-party NK-cell infusions are feasible, well tolerated, and associated with a negligible risk of GVHD, while potentially reducing leukemia relapse and enhancing antiviral immunity [6,13]. Consequently, multiple NK-cell platforms are under investigation, including cytokine-activated, ex vivo-expanded, cytokine-induced memory-like, CAR-engineered, and adaptive NK cells enriched for NKG2C expression [14,15,16,17]. Among these, adaptive NK cells have attracted particular interest owing to their enhanced effector function and long-term persistence.

Among human NK-cell subsets, adaptive (or memory-like) NK cells have emerged as a functionally distinct population with particular relevance for adoptive immunotherapy [17,18]. Although they are defined by a broader molecular program that includes epigenetic remodeling together with phenotypic features such as increased CD57 and self-specific KIR expression and reduced FcεRIγ, SYK and NKG2A expression [3,19,20,21], NKG2C remains one of the most widely used surrogate markers for identifying CMV-associated adaptive NK-cell populations in translational studies. These cells expand predominantly following CMV infection and have been associated with enhanced cytotoxicity, ADCC, persistence, and responsiveness to cytokine or target-cell stimulation compared with conventional NK cells [3,22]. Accordingly, CMV reactivation after allo-HSCT has consistently been associated with expansion of NKG2C+ adaptive NK cells and, in several studies, with a lower risk of AML relapse, suggesting a potential contribution to GVL activity [18,23,24].

Despite continuous improvements in automated GMP-compliant manufacturing platforms, donor-to-donor variability remains one of the least explored sources of manufacturing inconsistency for NK-cell products. Current optimization efforts have focused on manufacturing variables, such as culture conditions, cytokine combinations, and feeder-cell systems, whereas donor-related biological characteristics have received comparatively little attention. Because the frequency of NKG2C+ NK cells varies markedly among healthy individuals, baseline donor immunophenotype may represent an additional source of biological variability influencing manufacturing success. Identifying biomarkers associated with reproducible manufacturing could therefore contribute to donor qualification and improve manufacturing robustness within a Quality-by-Design (QbD) framework for advanced therapy medicinal products (ATMP).

We hypothesized that the baseline NKG2A/NKG2C immunophenotypic composition of healthy donors may influence the feasibility of GMP manufacturing of adaptive NK-cell products and may also affect the phenotypic characteristics of successfully expanded products. Accordingly, the primary objective of this proof-of-concept study was to evaluate whether representative healthy donors with NKG2C- or NKG2A-dominant NK-cell immunophenotypes differ in the feasibility of GMP manufacturing of adaptive NK-cell products. Secondary objectives were to explore the phenotypic, functional, and post-thaw characteristics of successfully manufactured products and to generate preliminary data supporting donor immunophenotype as a potential manufacturing biomarker.

## 2. Materials and Methods

### 2.1. Study Cohort and Donor Stratification

**Figure 1:**
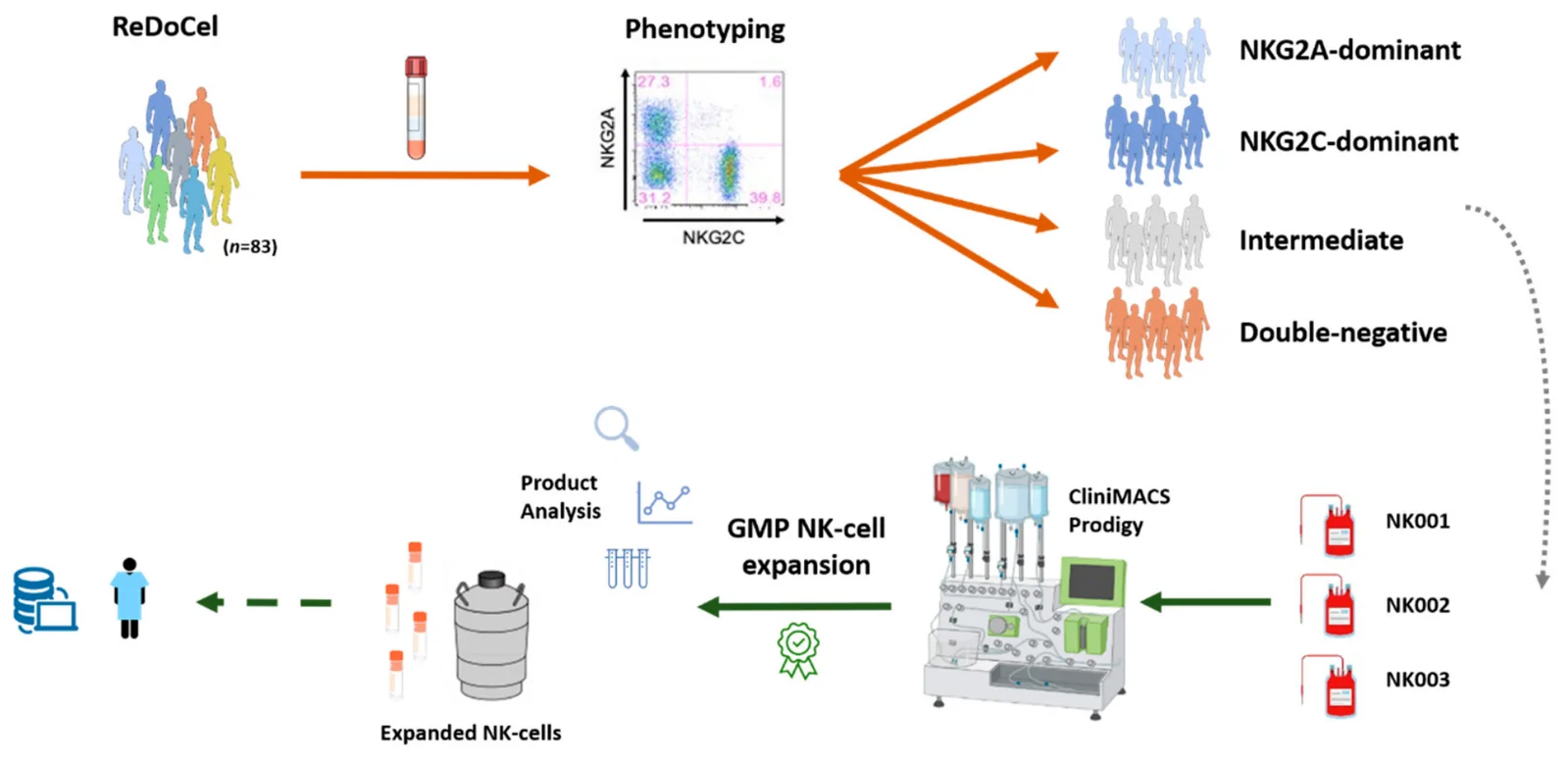
Overview of the study design. Healthy donors from the ReDoCel registry underwent peripheral blood NK-cell immunophenotyping, followed by donor stratification according to NK-cell subsets and selection for ex vivo expansion. Selected donors underwent GMP-compliant NK-cell manufacturing using the CliniMACS Prodigy® platform. Expanded NK-cell products were subsequently characterized for phenotype, cytotoxic function, and longitudinal post-thaw stability. The overall aim was to establish the foundation for a future GMP-compliant adaptive NK-cell bank to support off-the-shelf adoptive NK-cell therapies.

This multicenter preclinical study (Figure 1) included 83 healthy peripheral blood donors enrolled in the ReDoCel (Cell Donor Registry), established at the Valencian Community Transfusion Center (CTCV) [25]. Eligible donors were adults (≥18 years) who had undergone HLA typing by next-generation sequencing (NGS). The registry also included demographic characteristics, ABO/Rh blood group, viral serology (including CMV, and EBV), and routine hematological parameters collected as part of donor eligibility assessment.

Peripheral blood samples were randomly selected from the registry and immediately processed for immunophenotypic characterization by multiparametric flow cytometry (Supplementary Table S1). Hematopoietic populations and NK-cell subsets were analyzed using fluorochrome-conjugated monoclonal antibodies against CD16, CD56, NKG2A, and NKG2C. Samples were acquired on a FACSCanto™ II flow cytometer (BD Biosciences, San Jose, CA, USA) using FACSDiva software (v8.0.1), with at least 500,000 events collected per sample. Data were analyzed using FlowJo software (v10.5.3; BD Biosciences, San Jose, CA, USA).

Donors were subsequently stratified according to the frequency of NKG2A+ and NKG2C+ NK cells using unsupervised hierarchical clustering based on Euclidean distances and Ward’s minimum variance method (Ward.D2). Because both variables were expressed as percentages on the same measurement scale, no data standardization was applied before distance calculation. Ward’s method was selected because it minimizes within-cluster variance and generates compact clusters for continuous quantitative variables. The four-cluster solution was chosen based on dendrogram inspection, silhouette analysis, gap statistic estimation, and biological interpretability. As sensitivity analyses, k-means clustering (k = 4) and bootstrap resampling (1,000 iterations) were performed to evaluate clustering robustness and stability. A fixed random seed (set.seed(123)) was used for all stochastic procedures to ensure reproducibility. Statistical analyses were performed using R version 4.5.3 (RStudio version 2026.01.1+403), using the packages *cluster*, *mclust*, *fpc*, *factoextra*, *ggplot2*, and *dplyr*. This stratification guided donor selection for GMP manufacturing in the CliniMACS Prodigy® platform.

All participants provided written informed consent. The study was conducted in accordance with the Declaration of Helsinki and approved by the Ethics Committee of Hospital Clínico Universitario de Valencia (approval No. 2023/094).

### 2.2. GMP NK-Cell Manufacturing in the CliniMACS Prodigy® Platform

Expansion experiments were performed under GMP conditions at the Advanced Therapy Unit (*Unidad de Terapias Avanzadas*, UTA) of Hospital Universitario y Politécnico La Fe and at the GMP facility of Hospital Clínico de Valencia using a common GMP-compliant manufacturing protocol to facilitate future clinical translation. Manufacturing procedures were jointly validated by both GMP facilities, including standardized operating procedures, in-process controls, quality control assays, and product release criteria, to ensure methodological consistency and minimize inter-laboratory variability. Three donors representing distinct NKG2A/NKG2C immunophenotypic profiles were selected for GMP-compliant NK-cell manufacturing using the automated closed CliniMACS Prodigy® platform (Miltenyi Biotec, Bergisch Gladbach, Germany). One donor belonged to the NKG2C-dominant cluster, whereas two donors belonged to the NKG2A-dominant cluster.

Fresh buffy coats (<24 h after collection) were processed using the manufacturer’s PD-56 program for automated CD3^+^ T-cell depletion and CD56^+^ NK-cell enrichment. Purified NK cells were cultured in NK MACS medium supplemented with IL-2 (500 IU/mL), IL-15 (140 IU/mL), and IL-21 (100 ng/mL, added at culture initiation), all from Miltenyi Biotec, without feeder cells.

Cell cultures were maintained for 14 days; one manufacturing run was extended to 21 days because of insufficient expansion. During the first 7 days, cultures remained undisturbed. Thereafter, automated maintenance cycles were performed every 2–3 days using the CliniMACS Prodigy® platform, consisting of either a medium exchange or a volume reduction followed by replenishment with fresh cytokine-supplemented culture medium (feed). At each maintenance cycle, aliquots were collected for cell counting, viability assessment, culture adjustment, and immunophenotypic analysis.

Expansion efficiency was defined as the fold expansion of viable CD3-CD56+ NK cells relative to baseline. A threshold of 10-fold expansion was predefined as a process performance criterion for a successful manufacturing run. This threshold reflects the expansion required to obtain a clinically relevant cell dose under the present process and is not a product release specification. Manufacturing runs that failed to reach this threshold were considered suboptimal because the limited cell yield precluded comprehensive downstream characterization. The GMP release criteria established for the final NK-cell product were a CD3-CD56+ cell purity > 90%, with a minimum viability of 80%, and <5% residual CD3+ cells. 

### 2.3. Cryopreservation and Post-Thaw Stability Assessment

Final NK-cell products were cryopreserved in liquid nitrogen at a maximum concentration of 20 × 10^6^ cells/mL using a freezing media consisting of CryoStor® CS10 (Merck, Rahway, NJ, USA) and 20% human serum albumin (HSA; Grifols, Barcelona, Spain) mixed at a 1:1 ratio (final HSA concentration, 10%). Cell products were stored in either cryovials or cryogenic storage bags for subsequent longitudinal phenotypic and functional characterization for up to 9 months after cryopreservation to evaluate product stability.

### 2.4. Phenotypic and Functional Characterization

Expanded NK-cell products were characterized by multiparametric flow cytometry using a FACSLyric™ flow cytometer (BD Biosciences) to assess purity, phenotype, activation status, and functional competence. The expression of activating (DNAM-1, NKG2D, NKp30, and NKp46), maturation (CD57), and inhibitory/regulatory (KIR2DL1, KIR2DL2/DL3, PD-1, and TIM-3) markers was evaluated at baseline and at the end of culture. Details of the antibodies used are provided in Supplementary Table S1. Changes in receptor expression were calculated as log_2_ fold change (log_2_FC) using a pseudocount (ε = 0.1) to avoid zero values and were visualized as heatmaps.

NK-cell function was assessed by measuring CD107a degranulation together with intracellular IFN-γ and TNF-α production following 4 h of stimulation with K562 target cells or autologous PHA-activated blasts at an effector-to-target (E:T) ratio of 1:3 in the presence of BD GolgiStop^TM^ (monensin; BD Biosciences), according to the manufacturer’s instructions, and brefeldin A (10 µg/mL; Merck, Rahway, NJ, USA). Functional responses were normalized to assay-specific negative controls and expressed as the change from baseline to the final product (Δ = final − baseline). PMA (50 ng/mL; Merck) and ionomycin (1 µg/mL; Merck) were used as positive controls. Functional heterogeneity was further assessed by Boolean analysis of CD107a, IFN-γ, and TNF-α expression to identify mono-, bi-, and trifunctional NK-cell subsets.

### 2.5. Statistical Analysis

Categorical variables are presented as frequencies and percentages, whereas continuous variables are reported as mean ± standard deviation (SD) or median with interquartile range (IQR), according to data distribution assessed by the Shapiro–Wilk test. Statistical analyses were performed using RStudio (v2026.01.1+403). All tests were two-sided, and *p* < 0.05 was considered statistically significant.

Comparisons of CD56^dim^CD16^high^ and CD56^bright^CD16^dim^ subset frequencies within the NKG2A+ and NKG2C+ compartments were performed using the paired Wilcoxon signed-rank test. Differences in baseline NKG2A and NKG2C frequencies according to CMV serostatus were evaluated using the Wilcoxon rank-sum test. Differences in donor age among clusters were evaluated by one-way ANOVA and confirmed using the Kruskal–Wallis test when appropriate. Associations between donor clusters and categorical variables (sex, ABO blood group, and Rh factor) were assessed using Fisher’s exact test.

## 3. Results

### 3.1. Donor Immunophenotypic Profiling Identifies Distinct NKG2A/NKG2C Clusters

A total of 83 healthy peripheral blood donors were included. The median age was 33 years (range, 22–48), and 69% were female. The cohort showed heterogeneous distribution of ABO blood groups and HLA genotypes. CMV IgG seropositivity was detected in 82% of donors and EBV IgG in 95%.

Buffy coat enriched peripheral blood immunophenotyping showed that CD3+ T cells represented a median of 71.0% of total CD45+ leukocytes, with a predominance of CD4+ over CD8+ subsets (67.1% vs 29.6%, respectively). CD3-CD56+ NK cells accounted for a median of 10.75% of total CD45+ cells. Within the NK compartment, CD56^dim^CD16^high^ cells were predominant (89.7%), whereas CD56^bright^CD16^dim^ cells were a minor subset (4.25%). 

**Figure 2:**
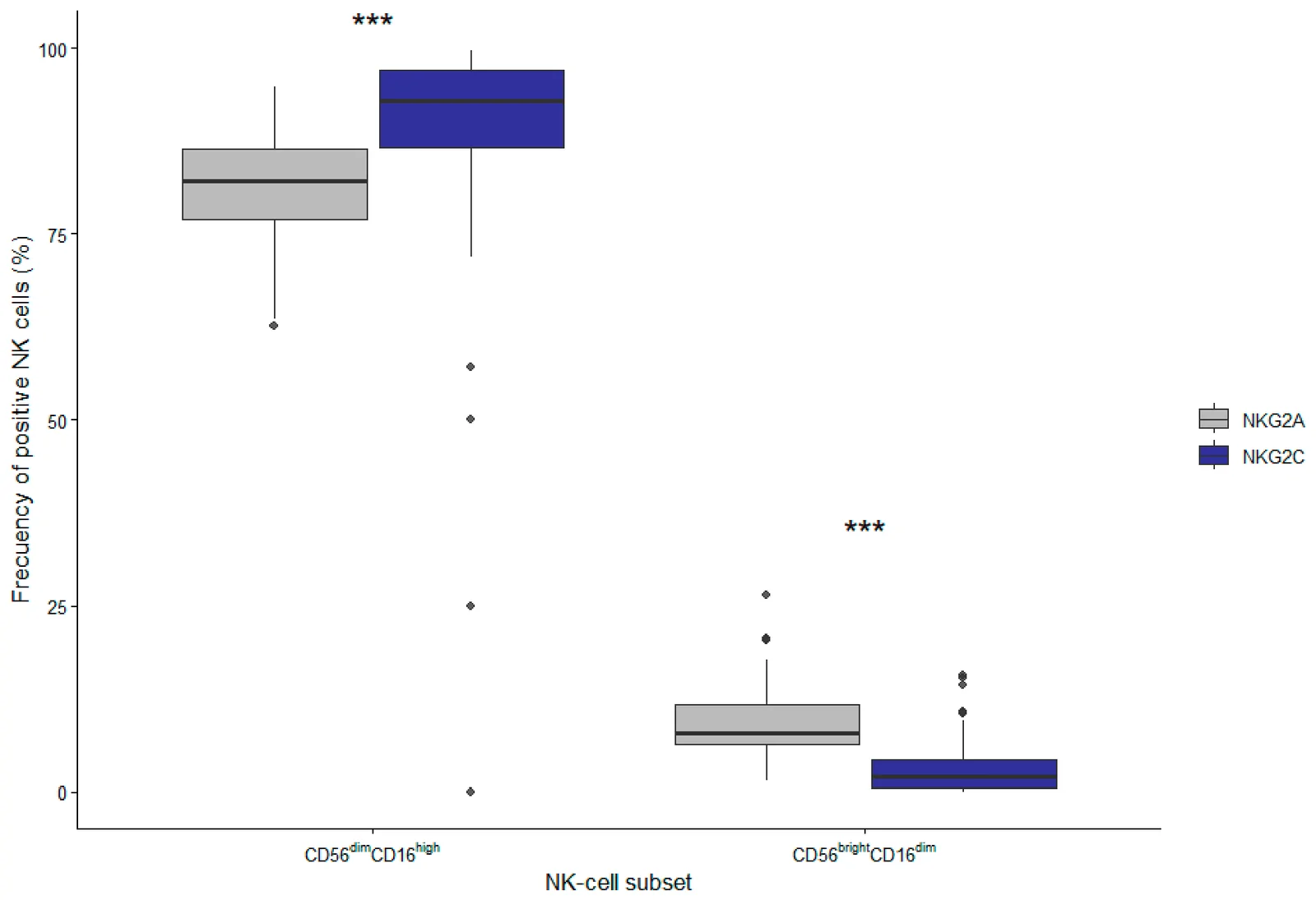
Distribution of NK-cell subsets according to NKG2A and NKG2C expression. Frequencies of the CD56^dim^CD16^high^ and CD56^bright^CD16^dim^ NK-cell subsets within the NKG2A+ and NKG2C+ compartments are shown. Each dot represents an individual donor, and box plots indicate the median and interquartile range (IQR). Statistical comparisons between NK-cell subsets within each receptor-defined compartment were performed using the paired Wilcoxon signed-rank test (*** *p* < 0.001).

The frequency of NKG2A+ and NKG2C+ NK cells was quantified in all donors and showed marked interindividual variability. Across the entire cohort, the median frequency of NKG2A+ NK cells was 39.3% (range, 12.3–75.4%), whereas the median frequency of NKG2C+ NK cells was 3.25% (range, 0.01–69.8%). Within the NKG2C+ NK-cell compartment, the CD56^dim^CD16^high^ cells predominated, whereas the CD56^bright^CD16^dim^ subset was preferentially represented within the NKG2A+ compartment (*p* < 0.001; Figure 2).

CMV-seropositive donors exhibited significantly higher frequencies of NKG2C+ NK cells than CMV-seronegative donors (median, 5.4% vs. 1.8%; Wilcoxon rank-sum test, *p* = 0.006), whereas NKG2A expression did not differ according to CMV serostatus (*p* = 0.80).

Unsupervised hierarchical clustering identified four donor groups based on NKG2A/NKG2C expression: NKG2A-dominant (*n* = 19), NKG2C-dominant (*n* = 12), intermediate (*n* = 42), and double-negative (*n* = 10). No significant associations were observed between cluster distribution and donor age, sex, CMV serostatus, ABO blood group, or Rh factor (Table 1). Although the mean silhouette coefficient indicated moderate cluster separation (0.37), sensitivity analyses supported the robustness of the classification. Hierarchical clustering showed good agreement with k-means classification (adjusted Rand index = 0.72), while bootstrap resampling demonstrated high stability for the NKG2A-dominant (Jaccard = 0.84) and NKG2C-dominant (Jaccard = 0.79) clusters, moderate stability of the intermediate cluster (0.68), and lower stability of the biologically heterogeneous double-negative cluster (0.51). This donor stratification was subsequently used for GMP manufacturing.

### 3.2. GMP-Compliant NK-Cell Manufacturing

#### 3.2.1. Manufacturing Performance Differs According to Donor Immunophenotype

Three GMP manufacturing runs (NK001-NK003) were performed using donors selected from the NKG2A- and NKG2C-dominant clusters, prioritizing individuals with the highest baseline frequencies of circulating NK cells. One NKG2C-dominant donor (NK001) and one NKG2A-dominant donor (NK002) were initially selected to compare manufacturing performance. Because NK002 showed suboptimal expansion, a second NKG2A-dominant donor (NK003) was subsequently manufactured to explore whether the limited expansion could have been related to donor-specific variability or to the NKG2A-dominant phenotype.

Initial PBMC counts ranged from 1.32 to 1.58 × 10^9^ cells, with viability > 96% (Table 2). Following CD3 depletion, residual CD3+ cell content ranged from 5.4 × 10^5^ to 1.35 × 10^6^ cells (0.07–0.19% of total PBMCs), corresponding to a median 3.07-log reduction (range, 2.89–3.32-log) in CD3+ cell content. Subsequent CD56 immunomagnetic enrichment recovered a median of 36.6% (range, 31.6–83.1%) of NK cells, yielding 60–80 × 10^6^ highly purified NK cells (median purity, 95.7%) with ≥ 99% viability. Residual CD3+ contamination after NK-cell enrichment ranged from 3.1 × 10^4^ to 1.6 × 10^5^ cells (0.05–0.27% of total selected cells) and was further reduced after ex vivo expansion, with final products containing 2.0 × 10^3^ to 7.9 × 10^5^ CD3+ cells (0.01–0.16% of total expanded cells).

Expansion performance differed substantially across manufacturing runs. NK001 achieved a final yield of 7.21 × 10^8^ NK cells after 14 days of culture, corresponding to a 13.1-fold expansion and successfully meeting the predefined GMP acceptance criterion (≥10-fold expansion). In contrast, NK002 and NK003 showed net cell loss relative to the seeded number (final yields corresponding to 0.6-fold and 0.3-fold of the input, respectively), resulting in insufficient cell yields for comprehensive downstream characterization. Consequently, some phenotypic and functional assays could not be completed or produced non-evaluable results.

#### 3.2.2. NKG2C-Dominant Manufacturing Generates Highly Activated Adaptive NK-Cell Products

**Figure 3:**
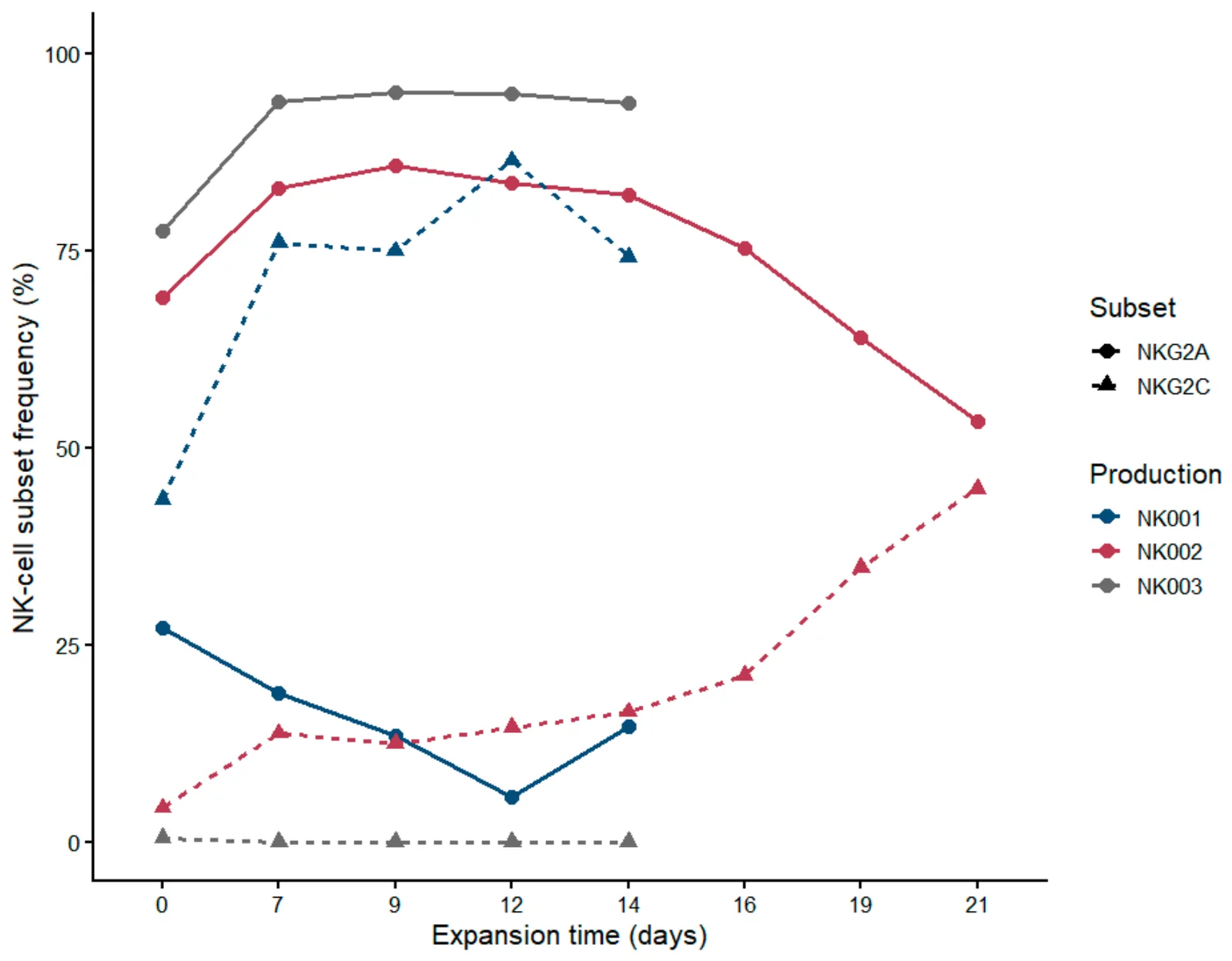
Dynamic remodeling of NKG2A+ and NKG2C+ NK-cell subsets during ex vivo GMP manufacturing. Longitudinal analysis of NKG2A+ (circles) and NKG2C+ (triangles) NK-cell frequencies during the three GMP manufacturing runs. NK001 is represented in blue, NK002 in red, and NK003 in grey. NK001 and NK003 were cultured for 14 days, whereas NK002 was cultured for 21 days.

Given differences in expansion performance, NK subset dynamics were evaluated (Figure 3). A consistent decrease in double-negative NK cells was observed across all runs. Double-positive cells remained stable in NK001 but declined in NK002 and NK003.

NK001 showed progressive enrichment of NKG2C+ NK cells throughout expansion, reaching 74.2% at the end of culture, with a reciprocal reduction in NKG2A+ cells to 14.7%. This pattern is consistent with a preferential expansion and/or selective maintenance of NKG2C-expressing NK cells under the evaluated culture conditions.

In NK002, initially classified as NKG2A-dominant, a dynamic remodelling of NK-cell subsets was observed over the course of expansion. An early increase in the NKG2A+ subset was detected during the first 14 days of culture, accompanied by a concomitant rise in NKG2C+ cells, indicating early phenotypic plasticity rather than a strictly NKG2A-restricted profile. Importantly, upon culture extension to day 21, a marked shift toward NKG2C+ predominance was observed, together with a significant reduction in NKG2A+ cells. Consequently, the final product displayed a more balanced immunophenotype, with 53.3% NKG2A+ and 44.8% NKG2C+ NK cells, consistent with a transition from a clearly NKG2A-dominant profile toward the intermediate cluster identified in peripheral blood.

In contrast, NK003 exhibited progressive NKG2A enrichment, with the final product comprising 93.7% NKG2A+ NK cells, whereas the NKG2C+ subset remained virtually absent (0.01%). Notably, the NKG2C+ subset accounted for only ~ 0.5% of the immunomagnetically enriched NK-cell population at culture initiation (following CD3 depletion and CD56 selection), which may have limited the detectable expansion of this subset under the evaluated conditions.

**Figure 4:**
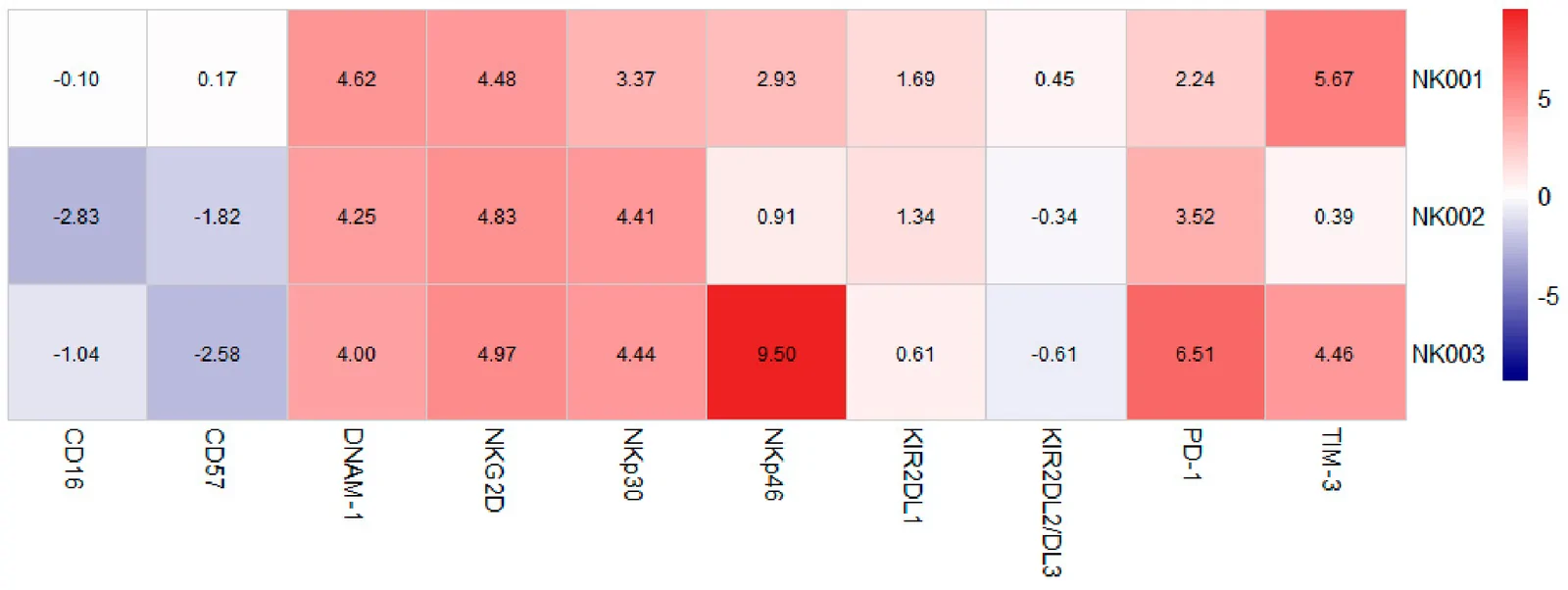
Changes in immunophenotypic receptor expression across CliniMACS Prodigy® manufacturing runs. Heatmap showing the log_2_ fold change (log_2_FC) in immunophenotypic marker expression between baseline and the final product for NK001, NK002, and NK003. Rows correspond to individual manufacturing runs and columns to the analysed markers. Color intensity reflects the magnitude and direction of expression changes relative to baseline, with positive values indicating upregulation and negative values indicating downregulation. Numerical values correspond to the log_2_FC of each marker.

Phenotypic characterization further demonstrated robust activation of the NK001 product (Figure 4). Activating receptors (DNAM-1, NKG2D, NKp30, NKp46) were markedly upregulated, with near-uniform expression of NKG2D and DNAM-1 after expansion. CD57 expression remained stable, whereas CD16 showed a slight decrease. Inhibitory receptors were largely stable, although TIM-3 and PD-1 increased, suggesting activation-associated regulation. Overall, activating signals predominated over inhibitory ones, with a higher mean log_2_FC across activating receptors than across inhibitory receptors (3.85 vs 2.51, respectively). These trends were reproducible in NK002 and NK003, although these products displayed lower CD57 and CD16 expression, suggesting a less differentiated phenotype.

**Figure 5:**
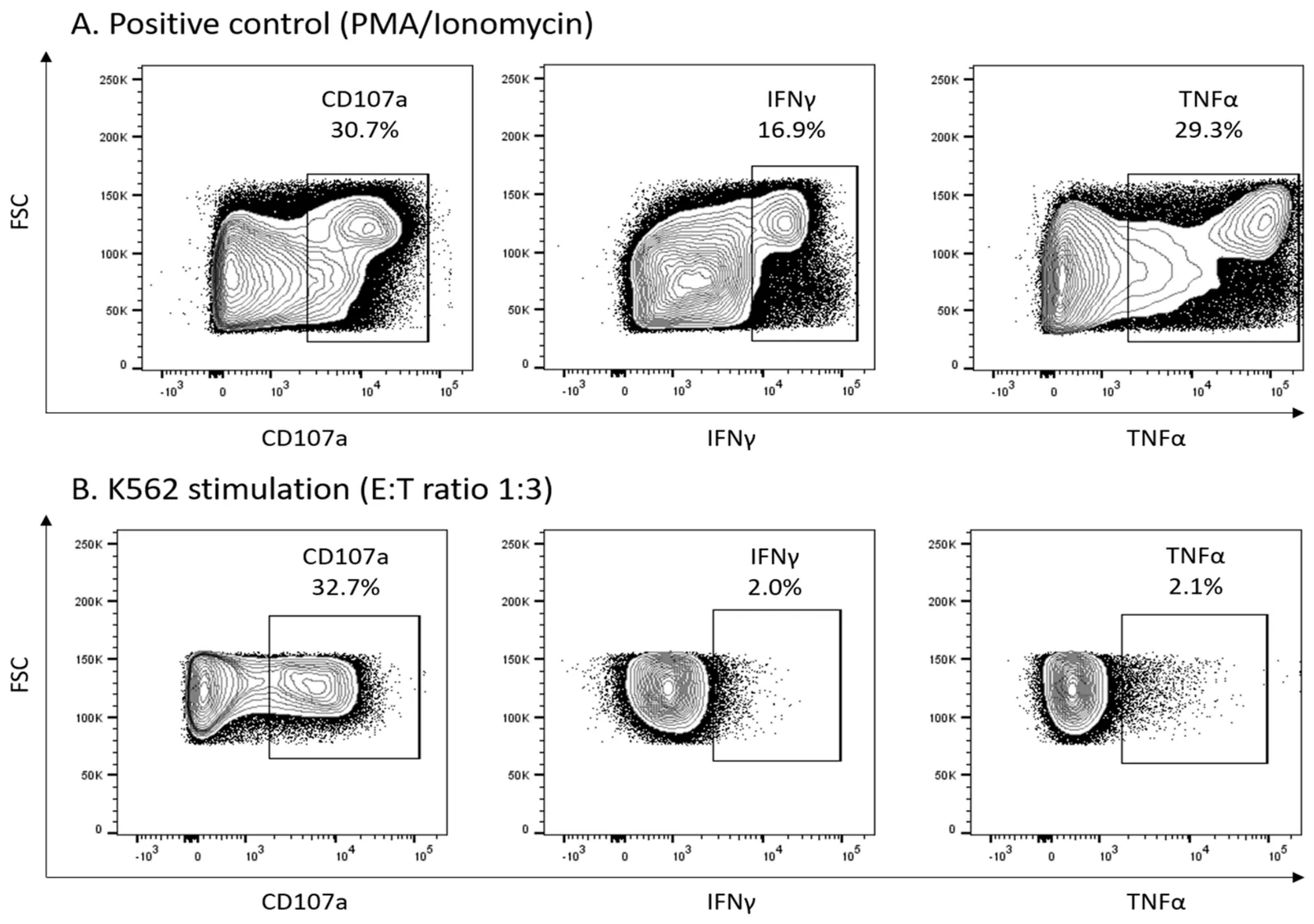
Representative FlowJo contour plots of NK-cell functional responses following NK001 expansion. Representative flow cytometry contour plots showing CD107a degranulation and intracellular IFN-γ and TNF-α expression in the final NK001 product after 4 h of stimulation with (**A**) PMA/ionomycin (positive control) or (**B**) K562 target cells (E:T ratio 1:3). Percentages indicate the proportion of positive cells within the gated CD56+ NK-cell population. Plots were generated in FlowJo using the Contour Plot display with the Show Outliers option enabled to improve visualization of low-frequency cell populations.

Functional assessment showed an increase in degranulation capacity in the NK001 product compared with its pre-expansion baseline (Figure 5). Expanded NK cells exhibited increased CD107a degranulation against K562 target cells (+6.5 percentage points over baseline), whereas IFN-γ and TNF-α production did not increase. In contrast, responses against autologous PHA-activated blasts remained at baseline levels, supporting preserved target specificity without evidence of increased autoreactivity. Boolean analysis confirmed a predominantly monofunctional profile driven by CD107a+ NK cells, with no increase in polyfunctional subsets under either stimulation condition. Functional assays in NK002 and NK003 were inconclusive due to low cell yield and variability.

#### 3.2.3. Cryopreservation Preserves Adaptive NK-Cell Phenotype and Function

Cryopreserved NK001 products were longitudinally evaluated at months +1, +3, +6, and +9 after storage in liquid nitrogen. Throughout follow-up, post-thaw viability remained ≥ 96% and NK-cell purity ≥ 90%. At month +9, the product retained its NKG2C-dominant phenotype, with 50.1% NKG2C+ and 17.1% NKG2A+ NK cells, while contamination by CD3+, CD14+, and CD19+ cells remained below 1% for each population. The expression of activating receptors was also preserved, with NKG2D, DNAM-1, and CD57 expressed on > 97% of NK cells. TIM-3 remained highly expressed (97.7%), whereas PD-1 was detected on 43.6% of cells. Overall, these phenotypic characteristics remained largely comparable to those observed immediately after expansion throughout the nine-month follow-up. Functional assessment at month +9 showed low basal degranulation and cytokine production against autologous PHA-blasts, whereas stimulation with K562 cells induced a marked degranulation response, with CD107a expression of 29.1%, accompanied by IFNγ and TNFα production of 6.86% and 8.23%, respectively, after correction for the corresponding negative controls. These findings indicate preservation of the phenotypic and functional characteristics of the NK001 product during cryopreservation for up to nine months. Longitudinal evaluation was not performed for NK002 and NK003 because of the limited cell yields obtained at the end of culture.

## 4. Discussion

NK-cell adoptive immunotherapy has emerged as a promising strategy to enhance immune reconstitution after allo-HSCT, particularly for preventing AML relapse and controlling CMV reactivation. Despite substantial advances in GMP manufacturing, donor-related factors influencing the quality and manufacturability of NK-cell products remain poorly characterized.

The first relevant finding was the marked interindividual variability in circulating NKG2A+ and NKG2C+ NK-cell frequencies. Consistent with previous reports, CMV-seropositive donors displayed significantly higher baseline NKG2C expression, whereas NKG2A levels were unaffected, reinforcing the role of NKG2C as a marker of CMV-driven adaptive NK-cell expansion [13,19]. Moreover, NKG2C expression was predominantly associated with the mature CD56^dim^CD16^high^ subset, while NKG2A was enriched within the CD56^bright^CD16^dim^ compartment. Because these subsets are classically linked to cytotoxicity and cytokine production, respectively, this distribution provides additional biological rationale for the hypothesis that NKG2C-enriched donors may differ in their manufacturing performance and in the phenotypic characteristics of expanded NK-cell products. Interindividual variability in NKG2C expression is largely determined by copy number variation at *KLRC2*: homozygous deletion abolishes NKG2C expression and heterozygosity reduces it, so that prior CMV exposure alone is not sufficient to generate a sizeable NKG2C+ adaptive compartment [18]. This may explain why only 12 of the 68 CMV-seropositive donors in our cohort were classified as NKG2C-dominant. *KLRC2* was not genotyped here, and the baseline NKG2C frequency of approximately 0.5% in the donor selected for NK003 would be compatible with, although not confirmatory of, a deletion carrier.

Hierarchical clustering identified four reproducible immunophenotypic donor groups based on NKG2A/NKG2C expression. Although cluster separation was moderate, concordance with k-means classification and bootstrap analysis supported the robustness of the NKG2A- and NKG2C-dominant clusters. These findings suggest that a simple immunophenotypic screening strategy may provide biologically meaningful information for donor stratification before GMP manufacturing. Serology alone therefore identifies prior exposure but does not predict the size of the NKG2C+ adaptive NK-cell compartment.

This proof-of-concept study suggests that the baseline NKG2A/NKG2C immunophenotypic profile of healthy donors may influence the feasibility of GMP expansion of NKG2C+ NK-cell products. Among three manufacturing runs performed, only the product generated from a donor enriched in NKG2C+ NK cells fulfilled the predefined manufacturing acceptance criteria (>10-fold expansion). The two NKG2A-dominant productions failed to achieve sufficient expansion, precluding subsequent complete functional characterization. Although this pattern is compatible with a potential association between NKG2C dominance and manufacturing success, the limited number of productions and absence of a successful replicate from an NKG2C-dominant donor prevent any causal or predictive conclusion. Moreover, donor-intrinsic characteristics cannot be considered independently of the manufacturing platform.

In this regard, the modest expansion observed in the present study should be considered in the context of the feeder-free manufacturing strategy employed. Although feeder-free approaches offer advantages for GMP translation by simplifying process standardization and avoiding concerns related to residual feeder cells, they may provide a less potent proliferative stimulus than feeder-based systems [26]. K562-derived feeder cells expressing membrane-bound IL-21, alone or in combination with 4-1BBL, have achieved substantially greater NK-cell expansion, reaching hundreds to several thousand-fold depending on the culture conditions [27,28,29]. Thus, the approximately 13-fold expansion achieved by NK001 and the failure of NK002 and NK003 to meet the predefined expansion criteria may have been partly influenced by the feeder-free platform, independently of the baseline NKG2A/NKG2C phenotype. Nevertheless, direct comparisons across manufacturing platforms should be interpreted cautiously because expansion is also influenced by donor characteristics, starting cell composition, culture duration, cytokine concentrations, and stimulation conditions. Taken together, these findings indicate that both donor biology and manufacturing design may contribute to expansion performance and should be considered when evaluating donor qualification strategies.

Expansion also induced dynamic remodeling of the NKG2A/NKG2C repertoire. As expected, the NKG2C-dominant donor became progressively enriched for adaptive NK cells throughout culture, whereas the donor with almost undetectable baseline NKG2C expression maintained an NKG2A-predominant phenotype. Interestingly, one initially NKG2A-dominant donor shifted toward an intermediate phenotype during prolonged culture. These observations are compatible with previous reports showing that ex vivo stimulation can preferentially enrich pre-existing adaptive-like NKG2C+ NK cells under selected culture conditions [26,30], while also indicating that this remodeling is not uniform across donors and may depend on the initial composition of the NK-cell compartment and other donor-specific factors. This is consistent with the hypothesis that expansion may preferentially amplify pre-existing adaptive NK cells rather than generate them de novo [31].

Beyond NKG2A/NKG2C remodeling, ex vivo expansion resulted in phenotypic changes consistent with an activated and differentiated NK-cell state, including increased expression of activating receptors such as NKG2D and DNAM-1 while largely preserving the inhibitory receptor repertoire [6]. TIM-3 was also consistently upregulated across manufacturing runs. Although traditionally considered an exhaustion-associated marker, TIM-3 expression in NK cells is context-dependent and may be induced by cytokine stimulation, particularly during ex vivo expansion, without necessarily indicating functional impairment [32,33]. The preservation of mature differentiation markers and KIR expression further supports the interpretation of the expanded product as differentiated and activated rather than intrinsically exhausted [3,33].

Functional assessment of NK001 was consistent with this activated phenotype. Ex vivo expansion increased CD107a-associated degranulation against K562 target cells compared with the pre-expansion baseline, while reactivity against autologous PHA-blasts remained limited. These findings are compatible with previous reports showing that ex vivo-expanded NK cells can acquire or enhance effector functions following cytokine- and feeder-mediated stimulation [27]. However, cytokine production was comparatively reduced and polyfunctional responses were limited. Importantly, because functional assays could not be completed for NK002 and NK003 owing to insufficient cell numbers, these findings represent the characterization of a single expanded product and should be interpreted descriptively rather than as evidence of superior functionality associated with NKG2C dominance. 

The successful production NK001 also demonstrated sustained post-thaw viability, phenotype and degranulation capacity during the nine-month follow-up period, supporting the feasibility of generating cryopreserved NK-cell products. This is relevant for the development of standardized, potentially off-the-shelf cellular therapies, although further studies are required to determine whether these characteristics translate into consistent in vivo persistence and antileukemic or antiviral activity. Automated closed platforms such as CliniMACS Prodigy® have demonstrated the feasibility of GMP-compliant NK-cell manufacturing, while highlighting ex vivo expansion as a major remaining challenge [11,34]. Our findings therefore suggest that donor immunophenotype may represent an additional, currently underexplored, source of manufacturing variability and could be investigated as a potential QbD attribute for donor qualification [12].

Several limitations should be considered when interpreting these findings. First, the donor screening strategy did not include a viability dye in the flow cytometry panel, which may have increased the risk of non-specific antibody binding to dead cells and potentially affected the accurate assessment of donor immunophenotypic characteristics. Second, this was a proof-of-concept study including only three GMP manufacturing runs, which does not allow definitive conclusions regarding the relationship between donor phenotype and manufacturing performance. In addition, the productions were performed across two GMP facilities, and with a single run per immunophenotypic group, donor-related biological variability cannot be formally distinguished from run-specific technical variability. Third, complete functional characterization could only be performed for the successfully expanded product because insufficient cell numbers were obtained from the remaining manufacturing runs. Fourth, no predefined potency threshold was established as a formal release criterion, and sterility and endotoxin testing were not performed because the products were generated exclusively for proof-of-concept research and were not intended for clinical infusion. These aspects will need to be addressed in future translational studies. Finally, adaptive NK cells were defined according to surface immunophenotype without incorporating complementary molecular, epigenetic or transcriptomic characterization, and the study did not evaluate in vivo persistence, antiviral efficacy or antileukemic activity.

Overall, these findings support the feasibility of using baseline NKG2A/NKG2C immunophenotyping as a potential approach for donor stratification before NK-cell manufacturing, while highlighting the importance of considering donor biology together with manufacturing conditions. The association between a NKG2C-dominant baseline phenotype and successful expansion in NK001 provides a biologically plausible hypothesis for further investigation but cannot be considered predictive based on the current dataset. Larger prospective studies including multiple donors across the different NKG2A/NKG2C phenotypic groups, standardized manufacturing conditions and, ideally, direct comparisons of feeder-free and feeder-based platforms will be required to determine whether donor immunophenotyping can improve manufacturing reproducibility and reduce production failures. If validated, this approach could ultimately contribute to donor qualification for the generation of standardized third-party NK-cell products for adoptive immunotherapy after allo-HSCT.

## 5. Conclusions

In conclusion, this proof-of-concept study suggests that the donor NKG2A/NKG2C immunophenotype may influence the feasibility of GMP-compliant expansion of adaptive NK-cell products. Manufacturing from a donor enriched in NKG2C+ NK cells yielded a stable, highly viable product that retained degranulation activity after cryopreservation. Although these preliminary observations require confirmation in larger manufacturing cohorts, they support further investigation of donor immunophenotyping as a biomarker to optimize donor selection and improve manufacturing consistency. Prospective studies integrating manufacturing and clinical outcomes will be needed to determine whether donor immunophenotype predicts successful production and contributes to the development of standardized off-the-shelf NK-cell therapies for allo-HSCT recipients.

## Figures and Tables

**Table 1:** Clinical and immunophenotypic characteristics of donor clusters defined by NKG2A and NKG2C expression.

Variable	NKG2A-Dominant Cluster	NKG2C-Dominant Cluster	Intermediate Cluster	Double-Negative Cluster	*p* Value
Donors, *n*	19	12	42	10	
NKG2A+ NK cells (%), *median* (*range*)	58.5 (52.7–75.4)	28.8 (12.7–40.3)	39.8 (22.2–58.9)	27.1 (12.3–30.6)	
NKG2C+ NK cells (%), *median* (*range*)	2.4 (0.8–10.1)	36.1 (27.7–69.8)	3.2 (0.0–26.9)	2.0 (0.0–4.8)	
CMV IgG positive, *n* (%)	15 (78.9)	11 (91.7)	34 (81.0)	6 (60.0)	0.368
Age (years), *median* (*range*)	37.0 (24–47)	31.5 (24–45)	34.5 (26–46)	36.5 (27–48)	0.242 (0.254)
Sex, *n* (%)					0.371
Female	11 (57.9)	8 (66.7)	29 (69.0)	9 (90.0)	
Male	8 (42.1)	4 (33.3)	13 (31.0)	1 (10.0)	
ABO blood group, *n* (%)					0.525
A	6 (31.6)	4 (33.3)	21 (50.0)	3 (30.0)	
B	2 (10.5)	0 (0.0)	4 (9.5)	1 (10.0)	
AB	0 (0.0)	1 (8.3)	1 (2.4)	1 (10.0)	
O	11 (57.9)	7 (58.3)	16 (38.1)	5 (50.0)	
Rh factor, *n* (%)					0.384
Rh-positive	15 (78.9)	9 (75.0)	37 (88.1)	7 (70.0)	
Rh-negative	4 (21.1)	3 (25.0)	5 (11.9)	3 (30.0)	

Donors were classified into four clusters according to the expression of NKG2A and NKG2C on circulating NK cells: NKG2A-dominant, NKG2C-dominant, intermediate, and double-negative. Demographic characteristics, CMV serostatus, ABO blood groups, Rh factor, and the frequencies of NKG2A+ and NKG2C+ NK cells are summarized for each cluster. Continuous variables are presented as median (range), and categorical variables as *n* (%). *p* values correspond to comparisons among the four clusters. For age, the *p* value from one-way ANOVA is shown first, with the corresponding Kruskal–Wallis *p* value in parentheses. Categorical variables were analysed using Fisher’s exact test. *p* values are not reported for NKG2A and NKG2C frequencies because these variables were used for cluster definition.

**Table 2:** Manufacturing characteristics and process outcomes of the three NK-cell productions performed using the CliniMACS Prodigy® system.

Variable	NK001	NK002	NK003
Initial PBMCs, *n* (viability, %)	1.58·10^9^ (99.3)	1.32·10^9^ (96.7)	1.51·10^9^ (96.8)
Post-CD3-depletion residual CD3+ cells, *n* (%)	7.1·10^5^ (0.10)	1.35·10^6^ (0.19)	5.4·10^5^ (0.07)
CD3 depletion, log	3.07	2.89	3.32
Post-CD56-enrichment CD3-CD56+ cells, *n* (%)	60·10^6^ (99.5)	80·10^6^ (95.7)	68·10^6^ (96.1)
CD3-CD56+ cells viability, (%)	99.9	99.7	99.5
Viable NK cells seeded, *n*	55·10^6^	35·10^6^	60·10^6^
Culture duration, days	14	21	14
Fold expansion	13.1	0.6	0.3
Final yield, *n*	7.21·10^8^	20·10^6^	20·10^6^

Data are presented as number of cells (*n*) and percentage (%) where indicated. The number of viable NK cells seeded refers to the number of cells used to initiate each expansion and may differ from the total number of CD3-CD56+ cells recovered after enrichment, as a fraction of the recovered cells was used for initial product characterization. PBMCs: peripheral blood mononuclear cells.

## Data Availability

The data presented in this study are available on request from the corresponding author. The data are not publicly available due to restrictions related to donor confidentiality under the ReDoCel registry.

## Supplementary Materials

The following supporting information can be downloaded at: https://www.mdpi.com/article/10.3390/cancers18172732/s1, Table S1: Flow cytometry antibodies used throughout the study. Antibodies used for donor screening, hematopoietic cell subset characterization, functional assays, and the analysis of activating and inhibitory NK-cell receptors are listed with their fluorochrome, clone, manufacturer, and catalog number.

## Abbreviations

The following abbreviations are used in this manuscript:

ADCC	Antibody-Dependent Cellular Cytotoxicity
Allo-HSCT	Allogeneic Hematopoietic Stem Cell Transplantation
AML	Acute Myeloid Leukemia
ATMP	Advanced Therapy Medicinal Products
CAR	Chimeric Antigen Receptor
CIK	Cytokine-Induced Killer
CMV	Cytomegalovirus
CTCV	Valencian Community Transfusion Center
EBV	Epstein–Barr Virus
GMP	Good Manufacturing Practices
GVHD	Graft-versus-Host Disease
GVL	Graft-versus-Leukemia
HAS	Human Serum Albumin
IFN-γ	Interferon gamma
IgG	Immunoglobulin G
IL	Interleukin
IQR	Interquartile Range
KIRs	Killer-cell Immunoglobulin-like Receptors
Log_2_FC	Log_2_ Fold Change
MDS	Myelodysplastic Syndromes
MHC-I	Major Histocompatibility Complex class I
NGS	Next-Generation Sequencing
NK	Natural Killer
PHA	Phytohemagglutinin
PMA	Phorbol 12-Myristate 13-Acetate
QbD	Quality-by-Design
ReDoCel	Cell Donor Registry
SD	Standard Deviation
TNF-α	Tumor Necrosis Factor-alpha

## References

[1] Döhner H., Wei A.H., Appelbaum F.R., Craddock C., DiNardo C.D., Dombret H., Ebert B.L., Fenaux P., Godley L.A., Hasserjian R.P. (2022). Diagnosis and management of AML in adults: 2022 recommendations from an international expert panel on behalf of the ELN. Blood.

[2] Carreras E., Dufour C., Mohty M., Kröger N. (2024). The EBMT Handbook: Hematopoietic Cell Transplantation and Cellular Therapies.

[3] Shimasaki N., Jain A., Campana D. (2020). NK cells for cancer immunotherapy. Nat. Rev. Drug Discov..

[4] Morvan M.G., Lanier L.L. (2016). NK cells and cancer: You can teach innate cells new tricks. Nat. Rev. Cancer.

[5] Stevens W.B.C., Netea M.G., Kater A.P., van der Velden W.J.F.M. (2016). Trained immunity: Consequences for lymphoid malignancies. Haematologica.

[6] Chen S., Zhu H., Journaidi Y. (2024). Comprehensive snapshots of natural killer cells: Functions, signaling, molecular mechanisms and clinical utilization. Signal Transduct. Target. Ther..

[7] Coënon L., Geincreau M., Ghiringhelli F., Villalba M., Bruchard M. (2024). Natural killer cells at the frontline in the fight against cancer. Cell Death Dis..

[8] Abbas A.K., Lichtman A.H., Pillai S. (2022). Cells of the innate immune system. Cellular and Molecular Immunology.

[9] Risso V., Lafont E., Le Gallo M. (2022). Therapeutic approaches targeting CD95L/CD95 signaling in cancer and autoimmune diseases. Cell Death Dis..

[10] Castro F., Cardoso A.P., Gonçalves R.M., Serre K., Oliveira M.J. (2018). Interferon-gamma at the crossroads of tumor immune surveillance or evasion. Front. Immunol..

[11] Min G.J., Kim N., Im K.I., Lee K., Kim T., Kim D.J., Cho S.G. (2026). Cytokine-induced killer cell therapy as a postremission strategy in high-risk patients undergoing autologous hematopoietic stem cell transplantation: A prospective investigator-initiated clinical study. Transplant. Cell. Ther..

[12] Fernández A., Navarro-Zapata A., Escudero A., Matamala N., Ruz-Caracuel B., Mirones I., Pernas A., Cobo M., Casado G., Lanzarot D. (2021). Optimizing the procedure to manufacture clinical-grade NK cells for adoptive immunotherapy. Cancers.

[13] López-Botet M., De Maria A., Muntasell A., Della Chiesa M., Vilches C. (2023). Adaptive NK cell response to human cytomegalovirus: Facts and open issues. Semin. Immunol..

[14] Herrera L., Santos S., Vesga M.A., Carrascosa T., García-Ruiz J.C., Pérez-Martínez A., Juan M., Eguizabal C. (2021). The race of CAR therapies: CAR-NK cells for fighting B-cell hematological cancers. Cancers.

[15] Siemaszko J., Marzec-Przyszlak A., Bogunia-Kubik K. (2021). NKG2D natural killer cell receptor: A short description and potential clinical applications. Cells.

[16] Vivier E., Raulet D.H., Moretta A., Caligiuri M.A., Zitvogel L., Lanier L.L., Yokoyama W.M., Ugolini S. (2011). Innate or adaptive immunity? The example of natural killer cells. Science.

[17] Siemaszko J., Marzec-Przyszlak A., Bogunia-Kubik K. (2023). Activating NKG2C receptor: Functional characteristics and current strategies in clinical applications. Arch. Immunol. Ther. Exp..

[18] Muntasell A., Pupuleku A., Cisneros E., Vera A., Moraru M., Vilches C., López-Botet M. (2016). Relationship of NKG2C copy number with the distribution of distinct cytomegalovirus-induced adaptive NK cell subsets. J. Immunol..

[19] López-Botet M., Muntasell A., Vilches C. (2014). The CD94/NKG2C+ NK-cell subset on the edge of innate adaptive immunity to human cytomegalovirus infection. Semin. Immunol..

[20] Hammer Q., Rückert T., Borst E.M., Dunst J., Haubner A., Durek P., Heinrich F., Gasparoni G., Babic M., Tomic A. (2018). Peptide-specific recognition of human cytomegalovirus strains controls adaptive natural killer cells. Nat. Immunol..

[21] Schlums H., Cichocki F., Tesi B., Theorell J., Beziat V., Holmes T.D., Han H., Chiang S.C., Foley B., Mattsson K. (2015). Cytomegalovirus infection drives adaptive epigenetic diversification of NK cells with altered signaling and effector function. Immunity.

[22] Gumá M., Angulo A., Vilches C., Gómez-Lozano N., Malats N., López-Botet M. (2004). Imprint of human cytomegalovirus infection on the NK-cell receptor repertoire. Blood.

[23] Mujal A.M., Delconte R.B., Sun J.C. (2021). Natural killer cells: From innate to adaptive features. Annu. Rev. Immunol..

[24] Björkström N.K., Strunz B., Ljunggren H.G. (2022). Natural killer cells in antiviral immunity. Nat. Rev. Immunol..

[25] Rudilla F., Carrasco-Benso M.P., Pasamar H., López-Montañés M., Andrés-Rozas M., Tomás-Marín M., Company D., Moya C., Larrea L., Guerreiro M. (2024). Development and characterization of a cell donor registry for virus-specific T cell manufacture in a blood bank. HLA.

[26] Chabannon C., Mfarrej B., Guia S., Ugolini S., Devillier R., Blaise D., Vivier E., Calmels B. (2016). Manufacturing natural killer cells as medicinal products. Front. Immunol..

[27] Jo H., Jo Y., Koh S.K., Lee M., Kim J., Kweon S., Park J., Kim H.Y., Cho D. (2025). Optimization of natural killer cell expansion with K562-mbIL-18/-21 feeder cells and assurance of feeder cell-free products. Ann. Lab. Med..

[28] Ojo E.O., Sharma A.A., Liu R., Moreton S., Checkley-Luttge M.A., Gupta K., Lee G., Lee D.A., Otegbeye F., Sekaly R.P. (2019). Membrane bound IL-21 based NK cell feeder cells drive robust expansion and metabolic activation of NK cells. Sci. Rep..

[29] Motallebnejad P., Kantardjieff A., Cichocki F., Azarin S.M., Hu W.S. (2023). Process engineering of natural killer cell-based immunotherapy. Trends Biotechnol..

[30] Liu L.L., Béziat V., Oei V.Y.S., Pfefferle A., Schaffer M., Lehmann S., Hellström-Lindberg E., Söderhäll S., Heyman M., Grandér D. (2017). Ex vivo expanded adaptive NK cells effectively kill primary acute lymphoblastic leukemia cells. Cancer Immunol. Res..

[31] Haroun-Izquierdo A., Vincenti M., Netskar H., van Ooijen H., Zhang B., Bendzick L., Kanaya M., Momayyezi P., Li S., Wiiger M.T. (2022). Adaptive single-KIR + NKG2C + NK cells expanded from select superdonors show potent missing-self reactivity and efficiently control HLA-mismatched acute myeloid leukemia. J. Immunother. Cancer.

[32] Ndhlovu L.C., Lopez-Vergès S., Barbour J.D., Jones R.B., Jha A.R., Long B.R., Schoeffler E.C., Fujita T., Nixon D.F., Lanier L.L. (2012). Tim-3 marks human natural killer cell maturation and suppresses cell-mediated cytotoxicity. Blood.

[33] Lieberman N.A.P., DeGolier K., Haberthur K., Chinn H., Moyes K.W., Bouchlaka M.N., Walker K.L., Capitini C.M., Crane C.A. (2018). An uncoupling of canonical phenotypic markers and functional potency of ex vivo-expanded natural killer cells. Front. Immunol..

[34] Córdoba-Espejo L., Sánchez-Vega L., García-Ortiz A., Castellano E., Oliva R., Ortiz-Ruiz A., Gil-Alós D., del Moral P., Fernández A., Sanjurjo D. (2026). GMP-compliant manufacturing of allogeneic peripheral blood CAR-NK cells for acute myeloid leukemia treatment. Cytotherapy.

