# Supplementary material for: Donor NKG2A/NKG2C Immunophenotype Influences the GMP-Compliant Manufacturing Potential of NK Cells for Adoptive Immunotherapy
Source: Cancers (Basel). 2026 Aug 23;18(17):2732. doi: 10.3390/cancers18172732 (PMC13564572; doi:10.3390/cancers18172732)
Supplement: Supplementary file 1 [file cancers-18-02732-s001.zip › cancers-4502262-supplementary.pdf]

Supplementary Table S1. Flow cytometry antibodies used throughout the study.

Antibodies used for donor screening, hematopoietic cell subset characterization, functional assays, and the analysis of activating and inhibitory NK-cell receptors are listed with their fluorochrome, clone, manufacturer, and catalog number.

S1A. Donor screening panel.

Marker	Fluorochrome	Clone	Manufacturer	Catalog No.
CD3	PerCP-Cy™5.5	UCHT1	BD Biosciences	560835
CD4	V500	RPA-T4	BD Biosciences	560768
CD8	APC-Vio® 770	REA734	Miltenyi Biotec	130-110-681
CD16	APC	REA423	Miltenyi Biotec	130-113-389
CD45	V450	HI30	BD Biosciences	560367
CD56	PE	REA196	Miltenyi Biotec	130-113-312
CD159a (NKG2A)	PE-Vio® 770	REA110	Miltenyi Biotec	130-113-567
CD159c (NKG2C)	Vio® Bright FITC	REA205	Miltenyi Biotec	130-117-707

S1B. Hematopoietic subpopulation panel.

Marker	Fluorochrome	Clone	Manufacturer	Catalog No.
CD3	FITC	HIT3α	BD Biosciences	555339
CD4	BB700	SK3	BD Biosciences	566392
CD8	APC-H7	SK1	BD Biosciences	560179
CD14	V450	MϕP9	BD Biosciences	560349
CD19	PE-Cy7	SJ25C1	BD Biosciences	557835
CD56	APC	B159	BD Biosciences	555518
Fixable Viability Stain 510	BV510	-----	BD Biosciences	564406

S1C. Functional assays panel.

Marker	Fluorochrome	Clone	Manufacturer	Catalog No.
CD56	PE	MY31	BD Biosciences	556647
CD107a	R718	H4A3	BD Biosciences	567036
CD159a (NKG2A)	PE-Vio® 770	REA110	Miltenyi Biotec	130-113-567
CD159c (NKG2C)	Vio® Bright FITC	REA205	Miltenyi Biotec	130-117-707
Fixable Viability Stain 510	BV510	-----	BD Biosciences	564406
IFNγ	BV421	B27	BD Biosciences	562988
TNFα	APC	MAb11	BD Biosciences	554514

S1D. Activating receptor panel.

Marker	Fluorochrome	Clone	Manufacturer	Catalog No.
CD16	BV786	3G8	BD Biosciences	563690
CD56	PE	MY31	BD Biosciences	556647
CD57	APC	NK1	BD Biosciences	560845
CD159a (NKG2A)	PE-Vio® 770	REA110	Miltenyi Biotec	130-113-567
CD159c (NKG2C)	Vio® Bright FITC	REA205	Miltenyi Biotec	130-117-707
CD226 (DNAM-1)	BV711	11A8	BD Biosciences	752659
CD314 (NKG2D)	BV605	1D11	BD Biosciences	743559
CD335 (NKp46)	BV421	9E2	BD Biosciences	564065
CD337 (NKp30)	R718	p30-15	BD Biosciences	752018
Fixable Viability Stain 510	BV510	-----	BD Biosciences	564406

S1E. Inhibitory receptor panel.

Marker	Fluorochrome	Clone	Manufacturer	Catalog No.
CD16	BV786	3G8	BD Biosciences	563690
CD56	PE	MY31	BD Biosciences	556647
CD158a (KIR2DL1)	APC	HP-3E4	BD Biosciences	564319
CD158b (KIR2DL2/DL3)	BV605	CH-L	BD Biosciences	743453
CD159a (NKG2A)	PE-Vio® 770	REA110	Miltenyi Biotec	130-113-567
CD159c (NKG2C)	Vio® Bright FITC	REA205	Miltenyi Biotec	130-117-707
CD279 (PD-1)	R718	EH12	BD Biosciences	566974
CD366 (TIM-3)	BV421	7D3	BD Biosciences	565562
Fixable Viability Stain 510	BV510	-----	BD Biosciences	564406
